# A curious abnormally developed embryo of the pill millipede ***Glomeris marginata*** (Villers, 1789)

**DOI:** 10.3897/zookeys.276.4767

**Published:** 2013-03-08

**Authors:** Ralf Janssen

**Affiliations:** 1 Uppsala University, Department of Earth Sciences, Villavägen 16, 75236 Uppsala, Sweden

**Keywords:** Teratology, Diplopoda, Development, Segmentation, vasa

## Abstract

This paper reports on an abnormally developed embryo (ADE) of the common pill millipede *Glomeris marginata*. This ADE represents a modified case of *Duplicitas posterior*, in which two posterior ends are present, but only one anterior end. While the major posterior germ band of the embryo appears almost normally developed, the minor posterior germ band is heavily malformed, has no clear correlation to the single head, little or no ventral tissue, and a minute amount of yolk. The anterior end of the minor germ band is fused to the ventral side of the major germ band between the first and second trunk segment. At least one appendage of the second trunk segment appears to be shared by the two germ bands. Morphology and position of the minor germ band suggest that the ADE may be the result of an incorrectly established single *cumulus* [the later posterior segment addition zone (SAZ)]. This differs from earlier reports on *Duplicitas posterior* type ADEs in *Glomeris marginata* that are likely the result of the early formation of two separate *cumuli*.

## Introduction

Abnormally developed myriapods have been reported repeatedly in the past (Balazuc and Schubart 1962). However, most of these specimens represent adult or earlier post-embryonic stages, and only very few studies investigate abnormal development during embryogenesis. The disadvantage of studying post-embryonic stages, including adults, is obvious: many of the malformations that occur during embryogenesis are lethal, and thus these specimens and their developmental abnormalities are never seen in such late developmental stages. Apart from that, the in-detail investigation of abnormal development in adults and earlier post-embryonic stages is often hampered by their complexity, while in embryonic stages the morphology of the abnormal germ band is much easier to interpret.

The majority of data on abnormally developed myriapods comes from centipedes (Selbie 1913, Minelli and Pasqual 1986, Kettle et al. 1999, Lesniewska et al. 2009) and symphylans (Juberthie-Jupeau 1962). Data from progoneate species including diplopods are rather scarce (Balazuc and Schubart 1962, Juberthie-Jupeau 1968, 1970, 1974, Hubert 1968, Ceuca 1989, Janssen 2011a, 2013). Most data on diplopods are known from the pill millipede *Glomeris marginata* (Villers, 1789) (Juberthie-Jupeau 1968, 1970, 1974, Hubert 1968, Janssen 2011a, 2013). The early development of this species is sensitive to variation in temperature, and development at high temperatures regularly leads to germ band duplications. Interestingly, all confirmed reports on germ band duplications in *Glomeris marginata* appear to be of the type *Duplicitas posterior*. That means that the germ band is composed of two posterior ends that share one anterior end (the head) (Janssen 2013). This kind of abnormality is likely a result of the formation/splitting of two *cumuli*, the posterior organization center(s), early during development (Janssen 2013).

This paper reports on the rare find of an abnormally developing embryo of *Duplicitas posterior* in which one of the posterior ends of the embryo is minute, lacking portions of its ventral ectodermal tissue, is detached from the yolk, and is atypically connected to the major germ band. The way in which the minor germ band is connected to the major germ band, and its point of connection, suggests that both posterior germ bands may have formed from one single (disturbed) posterior *cumulus*.

## Material and methods

### Animal husbandry, embryo collection, and fixation

Mature specimens of *Glomeris marginata* were collected in Germany (Nordrhein-Westfalen, Kreis Kleve) and cultivated in plastic boxes (22 cm × 13 cm × 5 cm) filled with decomposing leaves and moist clay. During egg deposition and embryogenesis the temperature was constantly held between 21–22°C. Eggs were manually separated from the clay egg-chambers and the chorion was removed by incubation in 2% sodium hypochloride for 1–2 minutes. Developing eggs were fixed for four hours in a mixture of 1 ml 4% formaldehyde in phosphate buffered saline pH 7.4 with 0.1% Tween-20 (PBST) and 1ml heptane. After fixation, embryos were washed in methanol and stored in methanol at -20°C for at least three weeks prior to in situ hybridization. The vitelline membrane was removed manually with fine forceps (Dumont No. 5, Fine Science Tools).

### Gene cloning

RNA isolation and cDNA synthesis were performed as per Janssen et al. (2004). A fragment of the *Glomeris marginata* ortholog of *vasa* was amplified via RT-PCR with the degenerate primers *vasa*_fw (GGN WSN GGN AAR CAN GCN GCN T) and *vasa*_bw (CK NCC DAT NCK RTG NAC RTA YTC). The fragment was cloned into a plasmid vector (pCRII-TOPO, Invitrogen). The sequence of the gene fragment was determined by sequencing (Big Dye Terminator Cycle Sequencing Kit; Perkin-Elmer Applied Biosystems, Foster City, CA, USA) chemistry on an automatic analyzer (ABI3730XL; Perkin-Elmer Applied Biosystems) by a commercial sequencing service (Macrogen, Seoul, Korea). The sequence is available in GenBank under the accession number HF543674.

### Whole mount in situ hybridization and nuclear staining

Cell nuclei were visualized with the fluorescent dye 4-6-Diamidin-2-phenylindol (DAPI). Embryos were incubated in 1 μg/ml DAPI in phosphate buffered saline with 0.1% Tween-20 (PBST) for 30 minutes. Excess DAPI was removed by washes in PBST for at least one hour. Whole mount in situ hybridization (WISH) was performed as described in Janssen et al. (2011).

### Data documentation

Pictures were taken with a digital camera (Axiocam; Zeiss, Jena, Germany) attached to a dissection microscope (Leica, Heerbrugg, Switzerland). Brightness, contrast and color values were corrected using image processing software (Adobe Photoshop CS2, V.0.1 for Apple Macintosh; Adobe Systems Inc. San Jose, CA, USA).

## Results and discussion

**Figure 1.**
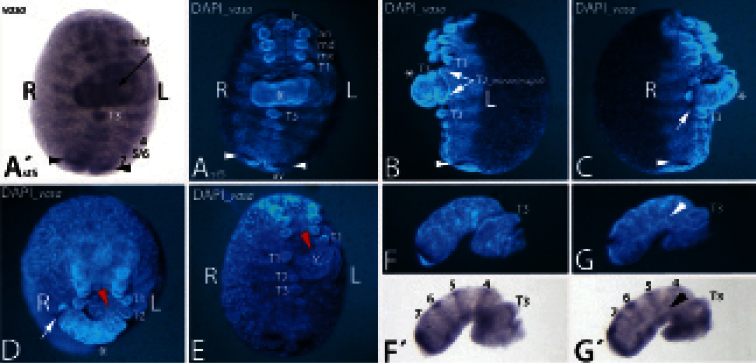
An unusual abnormally developed embryo of the type *Duplicitas posterior*. **A´–E** Whole-mount embryo. Anterior is towards the top. **F–G´** Separated minor germ band. Posterior is to the left. **A´** shows the bright-field photography of the embryo stained for *vasa*. Ventral view. Arrow points to stripe of expression in the minor germ band. **A** DAPI corresponding to A´. The asterisk marks the minor germ band. Arrowheads point to strong expression of *vasa* near the rear end. **B** Lateral view on left side. Asterisk and arrowhead as in **A**. Arrows point to T2 appendages. **C** Lateral view on right side. Asterisk and arrowhead as in **A**. Arrow points to strong expression of *vasa* in the minor germ band. **D** Ventral view. Embryo tilted towards beholder. Asterisk and arrow as in **C**. Red arrowhead points to contact point of minor germ band with major germ band. **E** Ventral view. Minor germ band was surgically removed. Red arrowhead as in **D**. **F/G** Separated minor germ band. Lateral views at different angles. **F´/G´** Bright-field photographs corresponding to **F/G**. Arrowhead in **G/G´** points to lack of dorsal *vasa* expression. Abbreviations: **4** to **7** *vasa* expression in the dorsal segmental units four to seven; **5/6** *vasa* expression in the fused dorsal tissue (diplosegment) aligned with T5 and T6; **an** antenna; **L** left side of embryo; **md** mandible; **mx** maxilla; **R** right side of embryo; **T1-T2** first to third walking limb; **Y** yolk.

A single abnormally developed embryo (ADE) with the here-described teratological morphology was found (Fig. 1). The specimen was stained for the DEAD-box helicase *vasa*. It represents a developing embryo at approximately stage 5 (staging after Janssen et al. 2004). The anomaly is principally of the type *Duplicitas posterior* with two separate posterior ends and a single anterior end. A variety of *Duplicitas posterior*-type embryos have been described for *Glomeris marginata* (Hubert 1968, Juberthie-Jupeau 1968, 1970, Janssen 2013). Although other germ band duplications, such as duplicated anterior regions (*Duplicitas anterior*) and completely or cross-wise duplicated embryos (*Duplicitas cruciata* and *Duplicitas completa*), have been described for *Glomeris marginata* (Juberthie-Jupeau 1968, 1970), these latter ADEs may indeed represent cases of *Duplicitas posterior* as well (discussed in Janssen 2013).

In the *Duplicitas posterior*-type embryo described here the development of the major germ band is almost normal, and its posterior part is clearly aligned with the single anterior pole (the complete head) (Fig. 1A–C). The development of the major germ band is abnormal in two respects. First, the posterior part of the major germ band is bent towards the embryo’s right side (Fig. 1A/A´). Second, all segments of the major germ band that are situated posterior to the contact point with the minor germ band are dorso-ventrally compressed, especially with respect to ventral tissue (Fig. 1A/E). This effect is more pronounced in tissue that is close to the contact point with the minor germ band. The limbs on the third trunk segment (T3), for example, are even fused and form a single broadened appendage (Fig. 1A/E). The limb buds of T4 and T5 stand unnaturally close together. The rear end of the major germ band, however, appears normal with a properly formed segment addition zone (SAZ), a pair of anal valves and an anus (Fig. 1A–E). Remarkably, while the amount of ventral tissue is reduced, the dorsal segmental units appear to have formed normally in the complete posterior part of the major germ band. This supports earlier findings that ventral and dorsal segmental patterning is decoupled (Janssen et al. 2004), and that ventral tissue can develop (or be maintained) in the absence of dorsal tissue (Janssen 2011a). Obviously, this embryo represents the opposite case in which dorsal tissue can develop independently from properly developing ventral tissue.

**Figure S1.**
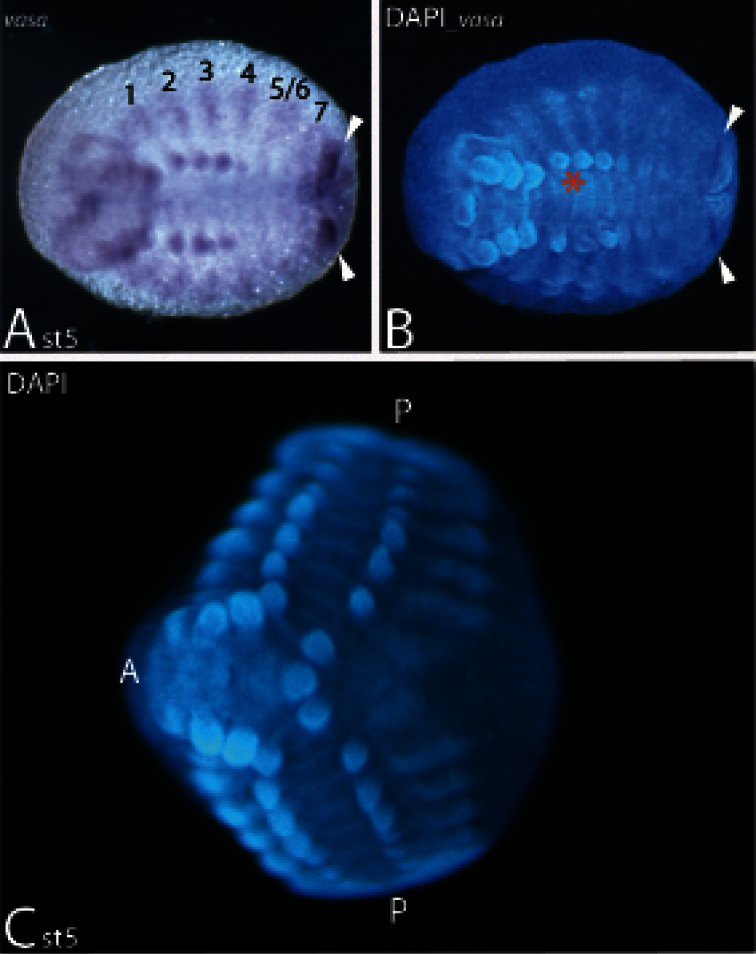
Expression of *vasa* (**A/B**) and a typical abnormally developed embryo (ADE) of *Duplicitas posterior* type (**C**).In all panels anterior is to the left. **A/B** Bright-field and corresponding DAPI-stained embryo. White arrowheads mark strong expression of *vasa* anterior to the anal valves. Red asterisk marks the position where the minor germ band is connected to the major germ band in the presented ADE (cf. Fig. 1). **C** Typical *Duplicitas posterior*-type embryo. Abbreviations: 1 to 7, expression of *vasa* in dorsal segmental units corresponding to ventral trunk segments one to seven; 5/6, first diplosegmental unit corresponding to ventral trunk segments five and six; **A** anterior pole (head); **P** posterior pole (anal valves).

The minor germ band is less well developed and lacks crucial parts of normally developing embryos (Fig. 1). It is fused to the left side of the major germ band between the first and the second trunk segments (Fig. 1B/E). The lateral view on the left side of the embryo reveals that at least one T2 appendage is shared by the two (major and minor) germ bands (Fig. 1B); otherwise a major germ band with four pairs (instead of three pairs) of primary outgrowing trunk limbs has to be assumed. Note that in *Glomeris marginata* the walking limbs of the first three trunk segments develop faster than the posterior walking limbs. Two pairs of developing limb buds are recognizable in the anterior region of the minor germ band. These represent the appendages of T3 and T4. The terminal region of the minor germ band ends in an unpaired region, which may represent the fused anal valves. This region lies posterior to the strong expression of *vasa* in the SAZ (Fig. 1F–G´). Unlike the situation in the major germ band, this domain of *vasa* expression is in a continuous transversal stripe, rather than with a ventral gap as in the major germ band and in normally developing embryos (Fig. S1). This implies that ventral tissue is lacking from the posterior end of the minor germ band. This assumption is further supported by the lack of the anus, and the fused anal valves. Tissue anterior to the SAZ may also comprise mainly dorsal tissue since in this area the expression of *vasa* is in continuous transverse stripes (Fig. 1F´/G´). In normally developing embryos *vasa* is strongly expressed in the mesoderm of dorsal segmental units, but only very weakly in the ventral mesoderm (Fig. S1). This indicates that ventral tissue may be lacking from most of the minor germ band. In the anterior part of the minor germ band, however, at least ectodermal ventral tissue must be present as indicated by the presence of the limb buds. The stripes of *vasa*-positive tissue in the minor germ band do not surround the complete ‘embryo’, but are discontinuous at the germ band’s dorsal side (Fig. 1G´). This means that dorsal closure has not yet happened. The number of *vasa* stripes in the minor germ band suggests that the same number of segments have formed in the minor and the major germ band. Four stripes of *vasa* can be seen posterior to T3 in the minor germ band (Fig. 1F´/G´). These stripes appear to correspond to three stripes of *vasa* posterior to T3 in the major germ band (Figs 1A and S1A). How is this possible? In *Glomeris marginata* dorsal segmental tissue corresponding to the ventral leg bearing segmental units T5 and T6 (and T7/T8) first develops as separate haplosegmental units, but subsequently the dorsal units fuse to form the diplosegments (Janssen 2011b). Apparently, in the minor germ band this fusion did not happen, and consequently two separate stripes (instead of one fused stripe) of *vasa* corresponding to T5 and T6 (stripes 5 and 6 in Fig. 1F´/G´) are still present. Since the development of ventral tissue is disturbed in the minor germ band, and dorsal fusion is apparently not taking place, the signal for dorsal fusion may thus originate from ventral tissue. In all hitherto described *Glomeris marginata* ADEs, except one specimen, the posterior ends of the embryos are normally developed (Janssen 2013). In the ADE described here, however, the posterior end of the minor germ band is clearly malformed with no anus, no or fused anal valves, and no ventral tissue, or at most only reduced amounts of it.

**Figure S2.**
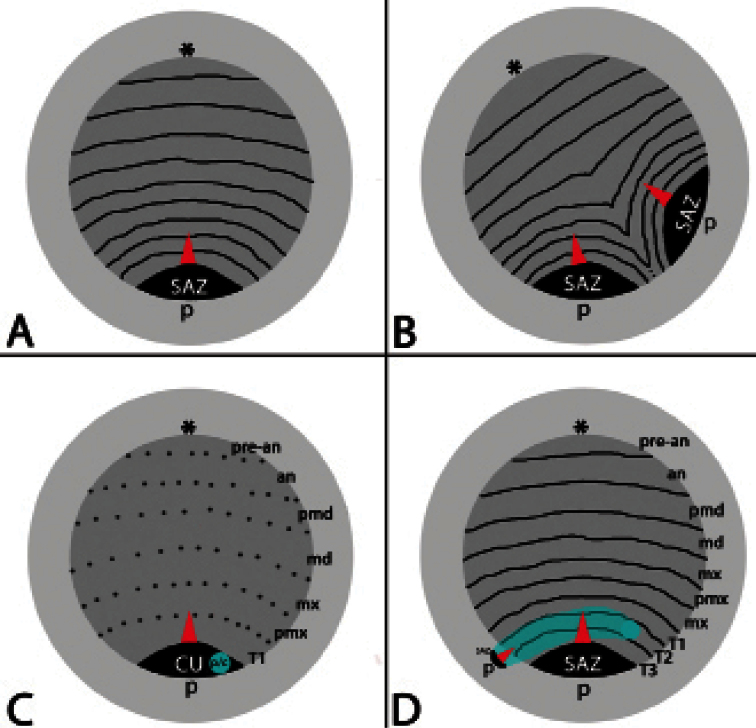
Schematic representation of normal development (**A**), ‘normal’ *Duplicitas posterior* type development (**B**), and the rare modified case of *Duplicitas posterior* reported in this paper (**C/D**). Light grey: yolk; dark grey: (major) germ band; transverse black lines: segmental boundaries; dashed lines: future segmental boundaries; light blue: minor germ band. Asterisks mark the anterior pole. Red arrowhead point to the direction in which new segments are added from the segment addition zone (SAZ). Abbreviations: **an** antennal segment; **c** cumulus of minor germ band; **cu** cumulus of major germ band; **md** mandibular segment; **mx** maxillary segment; **p/c** posterior (p) end of minor germ band and cumulus (c); **pmd** premandibular segment; pmx, postmaxillary segment; **pre-an** pre-antennal area; **SAZ** segment addition zone; **T1-T3** first to third trunk segment.

Among all described *Glomeris marginata* ADEs, this specimen is the only one that possibly developed two separate posterior ends from one posterior *cumulus*. In *Glomeris marginata* all segments posterior to T1 form from the SAZ that develops from the *cumulus*, and all segments anterior to (and including) T1 form from the blastoderm, the *regio germinalis* (Dohle 1964, Janssen et al. 2004). Thus the duplicated posterior germ band originates from the exact point where the *cumulus* was located prior to its transformation into the SAZ. The posterior pole of the minor germ band may thus represent a fraction of a single *cumulus* that split off early during development. This minor fraction was then only able to develop into the minor posterior germ band, and was not able to fully substitute for a complete *cumulus/*SAZ. Consequently, ventral derivatives have not been established properly. The position at which the minor germ band is connected to the major germ band supports the idea that its SAZ may represent a fraction of the major *cumulus*. The contact point of minor and major posterior germ bands is exactly at the transition of tissue from the anterior *regio germinalis* and tissue generated from the posterior *cumulus*/SAZ (Fig. S2C/D). In addition, the minor germ band connects to tissue within the major germ band, and not to the dorsal edge of the major germ band as would be the case if two separate *cumuli* were present (Figs S1C and S2A/B) (cf. Janssen 2013).
